# Structure and function of minor pilins of type IV pili

**DOI:** 10.1007/s00430-019-00642-5

**Published:** 2019-11-29

**Authors:** Theis Jacobsen, Benjamin Bardiaux, Olivera Francetic, Nadia Izadi-Pruneyre, Michael Nilges

**Affiliations:** 1 Structural Bioinformatics Unit, Department of Structural Biology and Chemistry, C3BI, Institut Pasteur, CNRS UMR3528, CNRS USR3756, Paris, France; 2 Sorbonne Université, Complexité du Vivant, 75005 Paris, France; 3 Biochemistry of Macromolecular Interactions Unit, Department of Structural Biology and Chemistry, Institut Pasteur, CNRS UMR3528, Paris, France

**Keywords:** Type IV pili, Minor pilins, Adhesion, Type II secretion system

## Abstract

Type IV pili are versatile and highly flexible fibers formed on the surface of many Gram-negative and Gram-positive bacteria. Virulence and infection rate of several pathogenic bacteria, such as *Neisseria meningitidis* and *Pseudomonas aeruginosa*, are strongly dependent on the presence of pili as they facilitate the adhesion of the bacteria to the host cell. Disruption of the interactions between the pili and the host cells by targeting proteins involved in this interaction could, therefore, be a treatment strategy. A type IV pilus is primarily composed of multiple copies of protein subunits called major pilins. Additional proteins, called minor pilins, are present in lower abundance, but are essential for the assembly of the pilus or for its specific functions. One class of minor pilins is required to initiate the formation of pili, and may form a complex similar to that identified in the related type II secretion system. Other, species-specific minor pilins in the type IV pilus system have been shown to promote additional functions such as DNA binding, aggregation and adherence. Here, we will review the structure and the function of the minor pilins from type IV pili.

## Introduction

Motility and pathogenicity of various bacteria are strongly dependent on proteinaceous appendages called pili, located at the cell surface [1, 2]. Pili are highly flexible, long fibers assembled in the envelope of bacteria. Several different types of pili have been identified and grouped based on their structure and assembly mechanism [3]. Both Gram-negative and Gram-positive bacteria can form pili, though the diversity of pili formed in Gram-negative bacteria is larger [3, 4]. In this review, we will focus on the type IV pili (T4P), which promote biological functions important for the pathogenicity of bacteria such as twitching motility, DNA uptake and adhesion to host cells [1]. The T4P family is generally subdivided into two groups, type IVa pili (T4aP) and type IVb pili (T4bP). This subdivision is based on the sequence of the pilins, the length of the leader peptide, and minor differences in their assembly [1, 5]. The organization of the genes required for pilus formation are different between T4aP and T4bP systems. T4aP genes are spread throughout the genome in several operons, whereas the T4bP genes are clustered in a single operon [2]. T4bP are involved in biofilm formation, bacterial colonization and cell adhesion [6]. T4aP have been identified as the molecular framework behind bacterial twitching motility and other functions such as DNA uptake [7]. Their flexibility, elongation and retraction allow them to ensure these different functions.

This review will highlight recent advances in the understanding of the assembly of T4P as well as in the biological functions and the structure of their minor pilins. Although the T4P assembly system is conserved between bacterial species, the nomenclature of T4P proteins is very heterogeneous. In this review, unless stated otherwise, we will use the nomenclature from *Pseudomonas aeruginosa,* one of the best-characterized T4P systems (Table 1).

**Table 1:** *P. aeruginosa* T4aP minor pilins and their homologs in both T4aP and T2SS from different organisms

T4a minor pilins			T2SS minor pseudopilins		
*Pseudomonas*	*Escherichia*	*Neisseria*	*Pseudomonas*	*Escherichia*	*Vibrio*
FimU	ppdA	PilH	GspH	XcpH	EspH
PilV	ppdC	PilI	GspI	XcpI	EspI
PilW	ppdB	PilJ	GspJ	XcpJ	EspJ
PilX	ygdB	PilK	GspK	XcpK	EspK
PilE	N/A	PilX/L PilV	N/A	N/A	N/A
PilY1	N/A	PilC2	N/A	N/A	N/A

## Building blocks of a pilus: the pilins

**Fig. 1:**
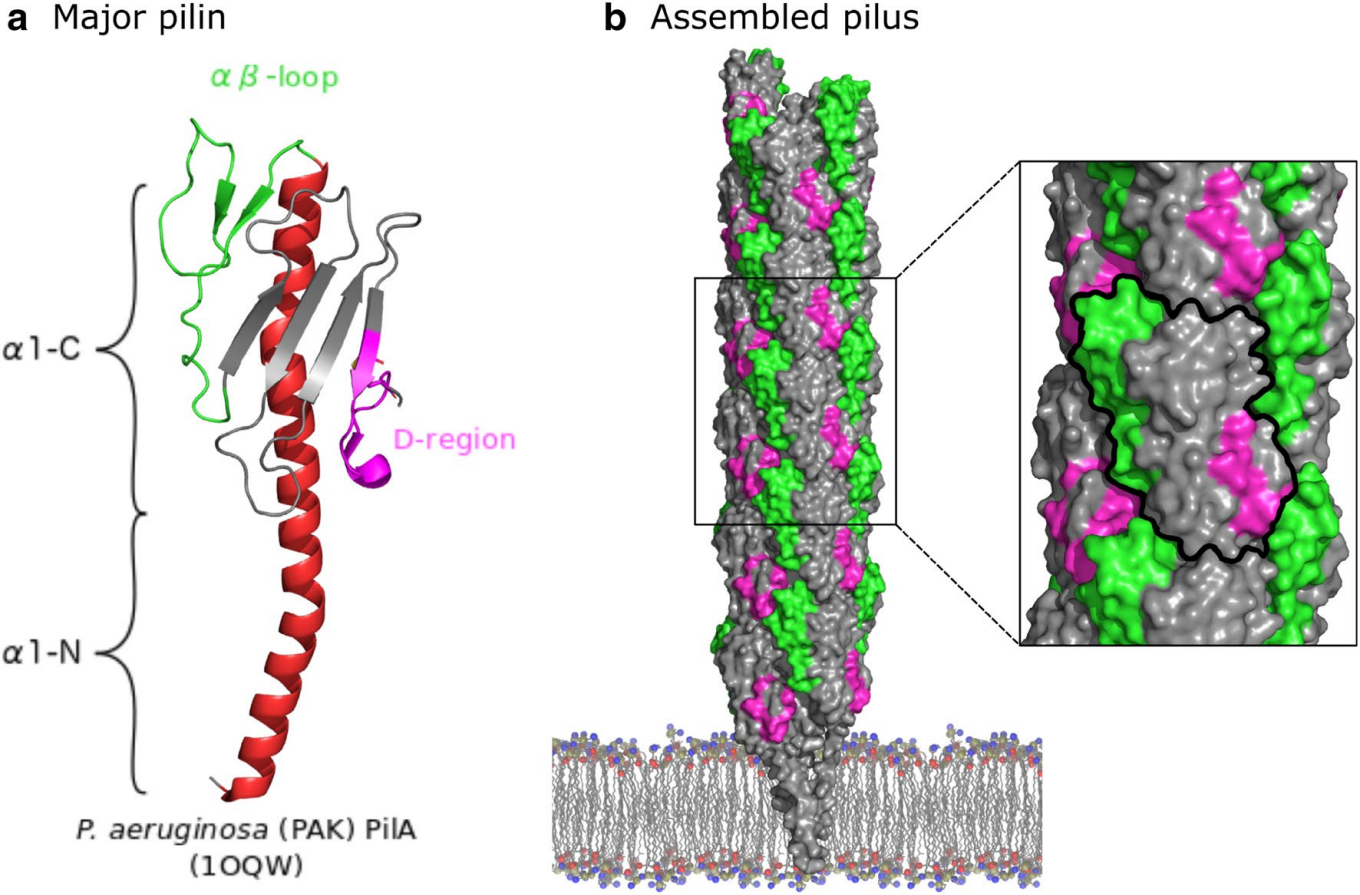
A major pilin and the type IVa pilus. **a** Molecular representation of the *P. aeruginosa* T4aP major pilin PilA (PDB:1OQW). The central α-helix is colored in red, the transmembrane N-terminal part (α1-N) and C-terminal (α1-C) part of the folded domain are indicated. The αβ-loop and D-region are highlighted in green and magenta, respectively. **b** The surface representation of the pilus assembled by subunits of PilA colored in gray and the αβ-loop and D-region are colored green and magenta, respectively (PDB: 5VXY). The zoom-in highlights the surface exposed of one PilA subunit in the pilus and the interactions between neighboring pilins

The major building blocks of a T4P are proteins called pilins, which are composed of a periplasmic domain and a transmembrane helix [3] (Fig. 1a). The bulk of a pilus is made up of thousands of copies of one subunit called the major pilin (Fig. 1b). Other pilins with a lower abundance, the minor pilins, have been shown to be essential for pilus formation and function [8]. Minor pilins can be further categorized into core and non-core minor pilins [9]. Core minor pilins are important for the formation of pili, despite their low abundance.

Pilins, both major and minor, are produced as prepilins with a short N-terminal leader sequence (signal peptide), which serves as an anchor to ensure correct insertion of the pilins in the cytoplasmic membrane [10]. The SecYEG translocon promotes prepilin membrane insertion in such a way that the N-terminal peptide is cytosolically exposed and the C-terminal domain of the pilins is exported to the periplasm (Fig. 1a) [12, 13]. The leader peptide is cleaved off by a specific prepilin peptidase to obtain the mature pilins that are competent for assembly [11].

**Fig. 2:**
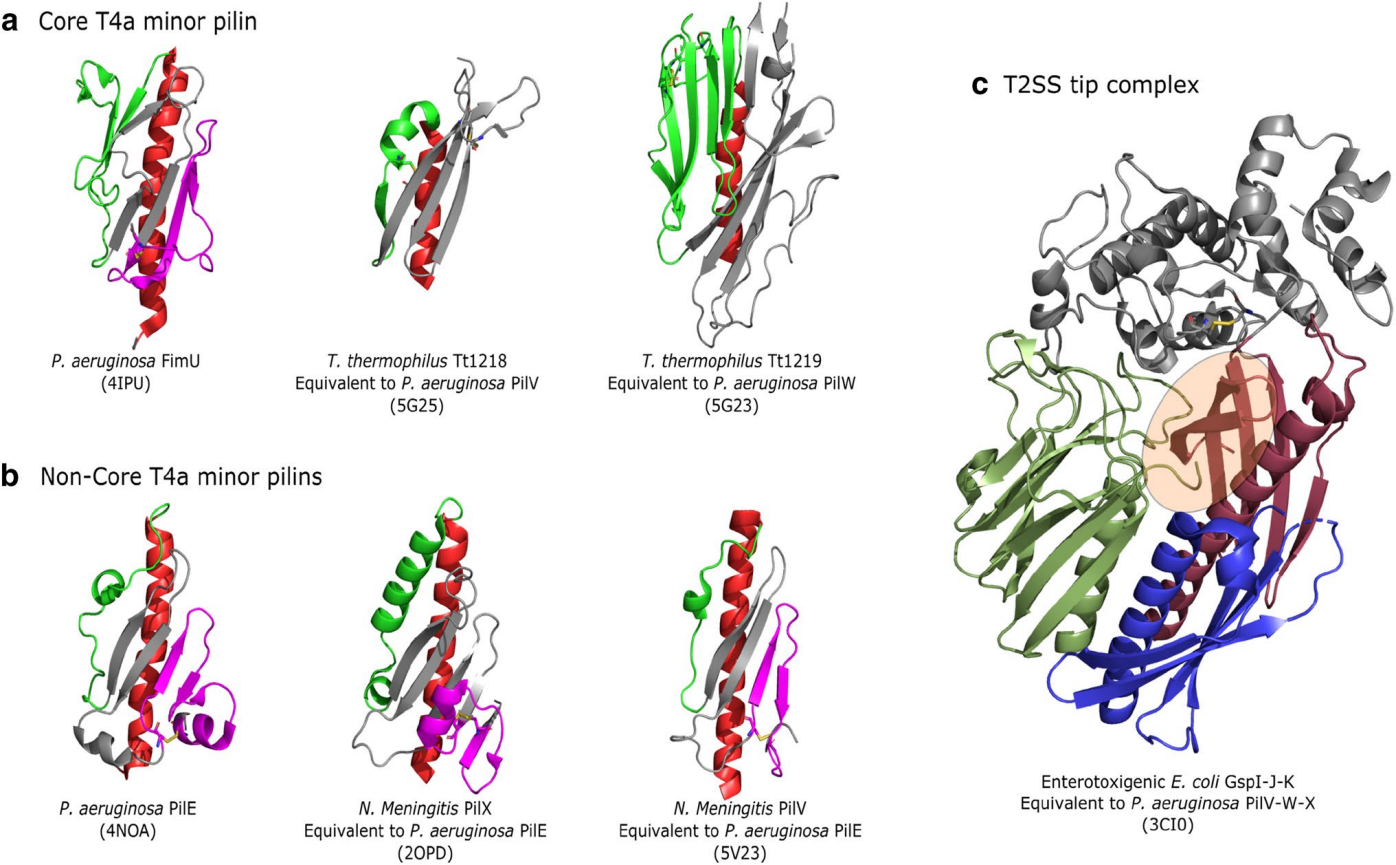
Comparison of minor pilins of T4aP from *P. aeruginosa* (or equivalent); the α1-helix (red), αβ-loop (green) and D-region (magenta) are highlighted. Disulphide bonds are represented as sticks. **a** Structures of core minor pilins of *P. aeruginosa* or equivalent pilins minor from *T. thermophilus*. We include these structures although the assignment as core minor pilins is indirect, by homology to GspI and GspJ. **b** Structures of non-core minor pilins of *P. aeruginosa* or equivalent minor pilins from *N. meningitis*. **c** Complex of three core minor pseudopilins of enterotoxigenic *E. coli*, GspI-J-K. GspI is colored in blue, GspJ is colored in green, the pilin domain of GspK is colored in brown and the inserted domain of GspK is colored in gray. An area covered by this inserted domain, which would form a solvent accessible cavity otherwise, is highlighted. Figure was prepared with The PyMOL Molecular Graphics System, Version 2.0 Schrödinger, LLC

The structural features of T4a pilins, both major and minor, are very similar (see Figs. 1a, 2a, b). They all adopt a lollipop-like shape with an N-terminal helix (α1), followed by a globular domain [9]. The α1 helix can further be separated into two segments, a hydrophobic N-terminal transmembrane (α1-N) and a C-terminal (α1-C) parts, which extends into the globular domain. Before assembly into the pilus, free pilins are anchored in the inner membrane by the transmembrane part (α1-N). At the C-terminal end of the transmembrane domain, the sequence contains a stretch of conserved residues usually limited by a glycine and a proline. In all known structures of T4P and the highly homologous type II secretion system (T2SS) major pilin subunits in the context of the pilus, this stretch disrupts the helix and forms an extended structure when incorporated into the fiber [14–17]. In the pilus, this region is also accessible to solvent as assessed by hydrogen–deuterium exchange in a T2SS pseudopilus [18] and a T4b pilus [19]. This conserved structural feature may enable the pilus to undergo spring-like extensions in response to shear forces [17]. Nothing is known about the structure of this region in minor pilins, since structural studies of individual minor pilins were limited to the soluble globular domains, and no structure of a complete pilus with a minor pilin is known.

The central axis of the globular domain is formed by the α1-C helix, which is surrounded by one or two antiparallel β-sheets composed of 4–7 strands, slightly rotated compared to the α-helical axis (Figs. 1a, 2a, b). Major structural differences between different minor and major pilins are restricted to the characteristic hypervariable regions: the αβ-loop connecting the α1-C helix to the β-sheet and the D-region enclosed by the C-terminal disulfide bridge found in some species. The αβ-loop is structurally very versatile and can contain various structural motifs. The ɑβ-loop of FimU includes distorted β-strands, whereas the D-region contains two antiparallel β-strands (Fig. 2a) [20]. Remarkably, the equivalents of PilV and PilW from *Thermus thermophilus*, Tt1218 and Tt1219, respectively, do not have a D-region, as the mostly conserved C-terminal disulfide bridge is absent in both proteins [21]. The C-terminal β-sheet has the same elongated shape in both pilins, the major difference is restricted to the αβ-loop. Tt1218 has a very short ɑβ-loop composed of only a short α-helix and a single β-strand. The ɑβ-loop of Tt1219 is much longer and contains a 5-stranded antiparallel β-sheet and two disulfide bridges.

## Assembly, elongation and retraction of pili

**Fig. 3:**
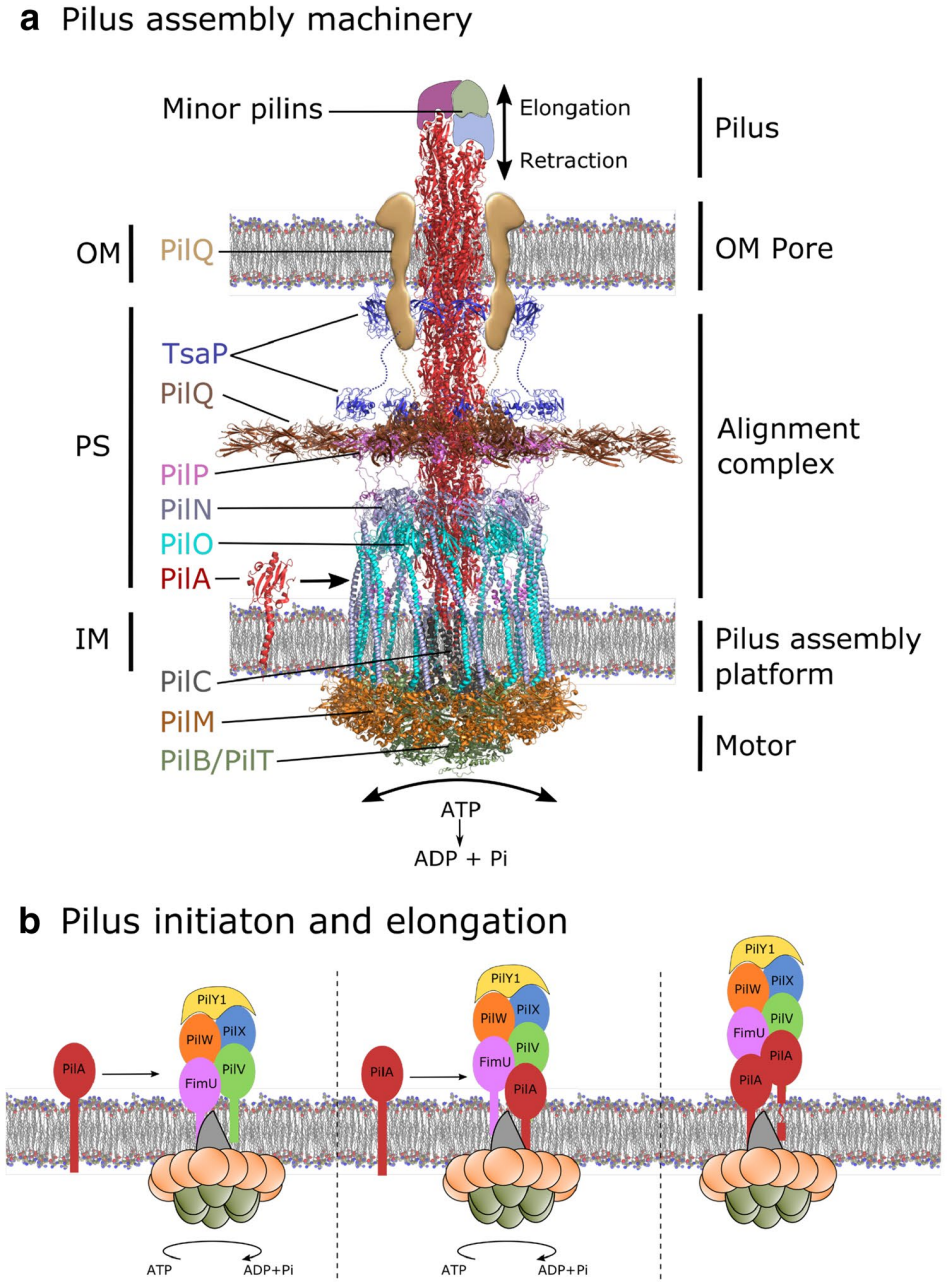
Type IVa pilus (T4aP) assembly. **a** The T4aP assembly machinery from *M. xanthus* (PDB:3JC8). On the left the outer membrane (OM), the periplasmic space (PS) and the inner membrane (IM) are marked. The individual proteins involved in the formation of T4P are shown and labeled. On the right the spanning of the different sub-complexes (pilus, OM pore, alignment complex, pilus assembly platform and motor) are indicated, see text for detailed description. **b** Schematic overview of the pilus initiation and elongation of tip complex. The orientations of the minor pilins are shown as hypothesized

The T4P assembly system is responsible for the elongation and the retraction of pili. In Gram-negative bacteria, the T4P assembly system is composed of between 10 and 18 different proteins forming complexes that are located in the inner membrane, in the periplasm and in the outer membrane [2]. Recent studies of the T4aP from *Myxococcus xanthus* [22] and *T. thermophilus* [23] by electron cryo-tomography have revealed the in situ organization of key elements in this assembly system that are summarized below (Fig. 3a) [22].

Elongation and retraction of the pilus are made possible by the addition or removal, respectively, of major pilin subunits (PilA) at the base of the pilus, and are facilitated by the central inner membrane platform protein, PilC and two antagonistic ATPase motors, PilB and PilT (Fig. 3) [24, 25]. PilC is in direct contact with the pilins and with the ATPases, which presumably rotate PilC to either elongate or retract the pilus [26, 27]. PilB and PilT occupy the center of the PilM cytoplasmic ring positioned near the inner membrane [28]. The PilM cytoplasmic ring interacts with the N-terminal transmembrane tail of PilN [28]. PilN forms a heterodimer with PilO, which assembles as a ring structure in the periplasm with the same stoichiometry as the cytoplasmic ring. The inner membrane protein PilP binds to this complex and connects it to the periplasmic domain of PilQ. The large PilQ multimer forms the secretin pore in the outer membrane necessary for the passage of the pilus through the membrane [29, 30]. Thus, the PilMNOP subcomplex links the cytoplasmic ATPases to the secretin pore [22, 31–33] and ensures that the motors of the assembly platform, PilC and PilB/PilT, are aligned with the secretin [34–36]. The fibers built by inserting pilins into the pilus base extend through the secretin pore out of the cell to perform their biological functions.

The T4P machinery is highly homologous to the type II secretion system (T2SS), in both assembly mechanism and pilus structure [37]. The sequence and structural organization of pilins of the two systems are conserved, and a T4P can be efficiently assembled by the T2SS [38]. The pilus, or the pseudopilus in T2SS, allows specific folded proteins in the periplasm to be secreted to the external medium. In T2SS, the minor pseudopilins promote assembly initiation [39] and have been implicated in substrate binding [40]. The absence of a retraction ATPase in the T2SS assembly platform is one of the major differences with the T4P system. The importance of calcium for the stability of the pseudopili from *Klebsiella oxytoca* suggests its implication in length control and passive disassembly of the pilus [14].

## Structure and function of core minor pilins

In the T4aP system, there are typically four core minor pilin genes that are clustered in an operon, which are homologous to the genes encoding four minor pseudopilins in the T2SS (Table 1). Deletion of these genes results in strong defects in pilus assembly and function, as demonstrated in *P. aeruginosa* [20] and enterohemorrhagic *Escherichia coli* (EHEC) [41]. In *N. meninigitidis,* deletion of each of the minor pilin genes—*pilH*, *I, J* and *K*—leads to a non-piliated phenotype [42]. Mutation in the ATPase *pilT* restores piliation, which led to the suggestion [43] that the role of these minor pilins is “anti-retraction”. However, each of them is required for fully efficient piliation, by analogy to the role of minor pseudopilins in the T2SS, suggesting that they are core minor pilins. A detailed study of the role of individual minor pilins in piliation and twitching motility demonstrated the role of the core minor pilins in assembly initiation, and showed that in the *pilT* mutant background, the residual piliation is dependent on minor T2SS pseudopilins [8, 44]. This confirms that T4a and T2SS minor pilins are functionally interchangeable, as had been shown before [45].

The homology between the four core minor pilins in T4P and T2SS led to the suggestion that they interact and form a complex responsible for the initiation of the pilus assembly in similar ways in both systems (Fig. 2c). The X-ray crystal structure of a complex of three minor pseudopilins, GspI, GspJ and GspK, from the T2SS of enterotoxigenic *E. coli* (ETEC) [46] serves as a structural template. In this complex, the tip subunit GspK acts as a cap, strongly suggesting that the core minor pilins are assembled first to form the tip of the nascent pseudopilus [46]. The minor pseudopilins form a staggered complex in the membrane, promoting initiation and setting the register for helical pseudopilus assembly [39].

This minor pilin complex would stabilize the tip of the pilus and provide a template for assembly of major subunits (Fig. 3b). In analogy to the T2SS, some of the minor pilins should differ both in sequence and structure from the major pilin to form a stable complex, which can remain anchored at the pilus tip. The GspI-J-K complex is composed of pilins of different molecular weight and three-dimensional shape (Fig. 2c), the central GspI being the smallest and GspK the largest, more than twice the size of the major pilin GspG. GspK has a large insertion in the globular domain, which might ensure that the complex is only incorporated at the tip, and its increased size can be imagined to shield the cavity that is formed by pilins of similar size and structure at the tip of a pilus (Fig. 2c). Unfortunately, there is no structure available for the GspK equivalent PilX from *P. aeruginosa,* nor for an equivalent T4P pilin from other species.

Although the sequence similarity makes it likely that a similar tip complex exists also in the T4P system, no structural information of this type of complex has been obtained to this date in the T4aP system. Remarkably, the large insertion present in GspK is absent in the T4P equivalent PilX from *P. aeruginosa*, and one has to postulate a different mechanism to cap the pilus. For example, in the T4bP from ETEC a single minor subunit CofB promotes initiation of assembly. Structural studies revealed that this subunit contains four distinct domains corresponding those of T4aP core minor pilins [47]. An interesting model based on another crystal structure proposes that CofB forms a homo-trimer that caps and primes pilus assembly [48]. Its homologue TcpB, the unique minor pilin in the *Vibrio cholerae* T4bP has been implicated in initiation of pilus assembly and disassembly [49]. Furthermore, recent biochemical evidence suggest that it forms a trimer localized at the pilus tip [50].

## Structure and function of non-core minor pilins

The non-core minor pilins have a more conserved fold and the diversity in the αβ-loop, and the D-region is more limited compared to the core minor pilins (see Fig. 2b). The αβ-loops of PilE [51] and the equivalents from *N. meningitidis* PilX [52] and PilV, are shorter and contain a small α-helical segment. The D-region in PilE and PilV from *N. meningitidis* both contain two β-strands, which are part of the conserved C-terminal β-sheet. Furthermore, PilE and PilX from *N. meningitidis* have a short helical segment in the D-region.

Non-core minor pilins are involved in the different biological functions that are promoted by T4P, including aggregation, adhesion and natural competence—the acquisition of external DNA (either inter or intra species) [7]. In *Neisseria* species, the minor pilin PilX promotes aggregation via pilus–pilus interactions [52], whereas PilV promotes adherence and signaling via receptors on eukaryotic cell surface [53]. In some species, additional proteins affect T4P-mediated adhesion. For example, PilY1 from *P. aeruginosa* is an adhesin which interacts with the complex formed by core minor pilins at the tip of the pilus [20]. PilY1 could also facilitate the adhesion to host epithelial cells. In addition, PilY1 and other minor pilins are involved in the regulation of *P. aeruginosa* virulence genes [54]. In EHEC no pilin-associated adhesins have been identified, but the expression of T4aP genes promotes twitching motility [55].

The minor pilin of *N. meningitidis* ComP binds DNA, recognizing a specific DNA sequence called the uptake signal [56] to promote natural transformation [57]. The DNA uptake mechanism mediated by T4P is not completely understood. However, it has been proposed that T4P play a role in the early step of DNA uptake [7]. A study of competence T4P from *Vibrio cholerae* by live fluorescence microscopy showed that DNA binds at the tip of the pilus [58]. This suggests that the binding of DNA is happening at the pilus tip principally via the minor pilins, despite their low abundance. ComP from the *Neisseria* species is also thought to be located at the pilus tip [59]. The retraction of the T4P brings the DNA to the cell surface where the DNA can be internalized via the secretin pore of the pilus or an alternate channel [7].

Some non-core minor pilins have large structural insertions. The recently determined structure of the minor T4a pilin ComZ from *T. thermophilus* [60]  shows an unusual structure with two domains, a pilin-like domain and a large additional domain inserted into its β-sheet. Hence, although not involved in pilus assembly, this minor pilin is likely located at the tip of the pilus, since the structural organization and the large size of this additional domain cannot allow upward incorporation of major pilins. Interestingly, this domain of ComZ was shown to be involved in DNA binding.

## Concluding remarks

With the emergence of antibiotic resistance, T4P are considered as a target for innovative antibacterial therapeutics [61]. T4aP of EHEC, among others, have been shown to be involved in biofilm formation, twitching motility and adherence [60]. The core minor pilins that likely form the tip of the pilus might play multiple roles, being at the same time responsible for the initiation of pilus formation, capping the pilus, and mediate specific biological functions. However, it is at the moment not clear if only the tip complex or also the major pilins can interact with surfaces or host cells. Further work is thus required in order to resolve the specific structural and biological mechanism of T4aP. Detailed structural knowledge of the minor pilins in the context of the entire fiber would be a major asset for the development of new vaccinal and therapeutic strategies.

## Abbreviations

T2SS: Type II secretion system
T4P: Type IV pilus
T4aP: Type IVa pilus
T4bP: Type IVb pilus
